# Learning to Look at the Bright Side of Life: Attention Bias Modification Training Enhances Optimism Bias

**DOI:** 10.3389/fnhum.2019.00222

**Published:** 2019-07-09

**Authors:** Laura Kress, Tatjana Aue

**Affiliations:** Department of Psychology, University of Bern, Bern, Switzerland

**Keywords:** attention bias modification training, cognitive bias modification, comparative optimism bias, expectancy bias, positive attention bias

## Abstract

Identifying neurocognitive mechanisms underlying optimism bias is essential to understand its benefits for well-being and mental health. The combined cognitive biases hypothesis suggests that biases (e.g., in expectancies and attention) interact and mutually enforce each other. Whereas, in line with this hypothesis, optimistic expectancies have been shown to guide attention to positive information, reverse causal effects have not been investigated yet. Revealing such bidirectional optimism-attention interactions both on a behavioral and neural level could explain how cognitive biases contribute to a self-sustaining upward spiral of positivity. In this behavioral study, we hypothesized that extensive training to direct attention to positive information enhances optimism bias. To test this hypothesis, for 2 weeks, 149 participants underwent either daily online 80-trial attention bias modification training (ABMT) toward accepting faces and away from rejecting faces or neutral control training. Participants in the ABMT group were instructed to click as quickly as possible on the accepting face among 15 rejecting faces randomly displayed on a 4-by-4 matrix; participants in the control group were instructed to click on the five-petaled flower depicted among 15 seven-petaled flowers. Comparative optimism bias and state optimism were measured *via* questionnaires before training, after one training week, and after two training weeks. ABMT enhanced comparative optimism bias, whereas control training did not. Our findings reveal that ABMT toward positive social information causally influences comparative optimism bias and may, thereby trigger the biases’ benefits for well-being and mental health. These results can (a) stimulate future neurophysiological research in the area of positive psychology; and (b) reveal an innovative low-cost and easy-to-access intervention that may support psychotherapy in times of rising numbers of patients with psychological disorders.

## Introduction

People are usually overly optimistic about their future (optimism bias; Weinstein, 1980) and preferably attend to positive information around them (attention bias; Pool et al., 2016). Both behaviors relate to benefits in everyday life (maintaining motivation) and clinical domains (protecting mental health; Joormann and Gotlib, 2007; Sharot, 2011). However, we know little about how optimism and attention bias interact (Kress and Aue, 2017). If we knew that the positivity biases mutually enforced each other (bidirectional interplay), instigating a self-perpetuating upward spiral of positive emotions (Garland et al., 2010), we could more easily employ the biases’ benefits in everyday life and clinical applications.

Theories such as the combined cognitive biases hypothesis suggest that cognitive biases (e.g., in expectancies and attention) interact and mutually enforce each other (Hirsch et al., 2006; Aue and Okon-Singer, 2015; Kress and Aue, 2017). From the combined cognitive biases hypothesis, we have recently proposed that optimism bias and positive attention bias dynamically interact and recruit a common underlying neural network. This network may comprise specific activations in the anterior and posterior cingulate cortices with functional connections to the limbic system (e.g., amygdala; see Kress and Aue, 2017, for further details). Furthermore, we proposed potential mechanisms of neural communication that might support the bidirectional interplay between optimism and positive attention bias.

Some of these theoretical considerations are supported by first empirical findings showing that optimistic expectancies indeed guide visual attention toward rewarding information (Kress et al., 2018). Large-scale neural networks comprising fronto-parietal brain regions in addition to the insula seem to underlie this mechanism (Kress et al., under revision). Notably, however, if bidirectional optimism-attention interactions exist, the reverse causal influence (and, later on, its underlying neural processes) must be demonstrated as well (Kress and Aue, 2017).

Attention bias modification training (ABMT: repeated training to attend to specific target stimuli and ignore others) may help investigators to study such causal influences of attention on optimism because it promises to modify attention (bias) and affect emotions (MacLeod and Mathews, 2012). Recent neural evidence suggests that ABMT reduces amygdala and insula activation toward emotional (threatening) stimuli (Månsson et al., 2013; Taylor et al., 2014). Furthermore, ABMT has been shown to increase frontal control and may thereby reduce anxiety symptoms (Browning et al., 2010; Taylor et al., 2014). Even though these results are promising, recent meta-analyses have revealed several methodological challenges related to ABMT (e.g., Cristea et al., 2015; Heeren et al., 2015b; Grafton et al., 2017; see Jones and Sharpe, 2017, for an overview).

Most studies in these meta-analyses used threat-avoidance ABMT to reduce pre-existing attention biases to threat in anxiety. Yet, these pre-existing biases are not consistently shown and can therefore not be modified in some ABMT studies (Mogg et al., 2017).

From the controversies concerning the appropriateness of threat-avoidance ABMT, a novel approach (positive-search ABMT) has been considered more promising in eliciting beneficial emotional outcomes: Positive-search ABMT works more reliably in home settings and elicits emotional benefits without exclusively relying on changes in attention bias (Mogg et al., 2017). Furthermore, the process trained in positive-search ABMT (i.e., finding a positive stimulus among negative stimuli) may be more adaptive and transferrable to real-life situations than processes trained in traditional threat-avoidance ABMT (e.g., reacting to a dot appearing after a neutral stimulus). For instance, the particular positive-search ABMT used in the current behavioral study has been developed to improve people’s ability to inhibit social rejection and approach social acceptance information by training them to find the smiling face in a crowd of frowning faces (Dandeneau and Baldwin, 2004). This training to direct attention to adaptive information may be most effective to boost optimism bias.

People’s attention was biased away from negative and toward positive social information after completing positive-search ABMT in most (Dandeneau and Baldwin, 2004, 2009; Dandeneau et al., 2007; Waters et al., 2013; De Voogd et al., 2014, 2016) but not all (Waters et al., 2015) studies assessing attentional changes following training. More important, positive-search ABMT elicited diverse beneficial emotional outcomes (lower perceived stress, enhanced self-esteem/positive self-regulation: Dandeneau et al., 2007; Dandeneau and Baldwin, 2009; reduced anxiety/social phobia: Waters et al., 2013, 2015, 2016; De Voogd et al., 2014; but see De Voogd et al., 2016, for null findings). These beneficial outcomes, in turn, are also associated with optimism bias for positive future events (e.g., self-esteem and self-regulation; Hoorens, 1996; Armor and Taylor, 1998). Thus, positive-search ABMT constitutes a promising tool to examine the effects of positive attention processes on optimism bias.

For two reasons, we decided to focus on optimism bias for positive future events in the current study, both relating to evidence in the literature that optimism biases for positive and negative future events represent different aspects with independent motivating factors (Weinstein, 1980; Hoorens, 1996). First, self-enhancement has been suggested to be an important motivating factor for optimism bias for positive future events but not for optimism bias for negative future events (which may be related to different motivating factors such as impression management; Hoorens, 1996). The positive-search ABMT used in the current study has been developed to enhance positive social cognition and self-regulation, thereby permitting the examination of cognitive mechanisms of self-enhancing positivity. Second, the positive-search ABMT used here has shown to enhance self-esteem (Dandeneau et al., 2007; Dandeneau and Baldwin, 2009). Because higher self-esteem was particularly associated with elevated optimism bias for positive future events but less so with optimism bias for negative future events (Hoorens, 1996), we hypothesized that the positive-search ABMT may be particularly effective in enhancing optimism bias for positive events.

The present work investigates whether repeatedly directing attention toward positive or away from negative social information during training causally influences optimism bias. Participants were randomly assigned to ABMT or control training. Before training, after one training week, and after two training weeks, all participants completed the Comparative Optimism Scale (COS; Weinstein, 1980; measuring optimism bias *via* social comparison) and the Future Expectancy Scale (FEX; Peters et al., 2015; measuring current optimistic states that are not necessarily biased but likely instigate optimism bias; see Garland et al., 2010, for details on how momentary emotional experiences trigger durable changes in emotional systems/affective styles). Because optimistic states vary across situations, ABMT that directs attention to positive aspects of a situation may trigger such state optimism.

Whereas comparative optimism bias was measured to uncover the importance of social/self-enhancing components in relation to attention processes, state optimism was measured to examine whether attention processes elicit optimistic states that then instigate the biases’ formation. By measuring these different aspects, the current study can uncover crucial determining factors for the influence of attention processes on optimism bias (social comparisons and/or transient optimistic states). If repeatedly directing attention to positive information through training enhances optimism bias, then people’s level of comparative optimism bias and/or state optimism should increase after participating in positive-search ABMT but not after the control training.

Even though prior literature proposes that ABMT affects people’s responses to emotional and motivational cues (Beard et al., 2012)—such as when forming expectations about positive, motivationally salient future events—ABMT does not seem to directly affect people’s mood (Dandeneau and Baldwin, 2004; Dandeneau et al., 2007; Beard et al., 2012). To replicate this finding and rule out the possibility that potential training effects on optimism bias arose because of changes in mood, we assessed participants’ mood with the Positive and Negative Affect Schedule (PANAS; Watson et al., 1988) as a secondary outcome in the current study.

## Materials and Methods

### Participants

From a recent systematic review of meta-analyses on the efficacy of ABMT on emotional outcomes, we anticipated a small effect of ABMT on optimism bias (Jones and Sharpe, 2017; because effect sizes varied considerably, we chose the most modest assumption of a small effect). A minimum sample size of 128 to detect such small effect ($\eta_{\text{p}}^{2}$ = 0.02) was determined with a power analysis (*α* = 0.05, power = 0.95). Because we expected high dropout rates over the two training weeks, 20 additional participants were tested. Thus, 149 healthy participants with normal or corrected-to-normal vision, who did not report using psychoactive substances, took part in this online study. Sixteen participants were excluded from data analysis because of technical errors in data logging (*N* = 2), or because they did not complete the training on more than 2 days (*N* = 14), leaving a final sample of 133 participants (experimental group: *N* = 71, 26 male, age: *M*_Exp_ = 22.17 years, *SD*_Exp_ = 3.92 years; control group: *N* = 62, 16 male, age: *M*_Con_ = 23.35 years, *SD*_Con_ = 3.16 years). Participants were randomly assigned to a group and did not show baseline differences in any of the reported outcome measures (i.e., optimism or mood; all *ps* ≥ 0.283). However, the experimental group displayed slightly lower trait optimism scores than the control group did (i.e., Life Orientation Test-Revised (LOT-R) sum scores; Scheier et al., 1994: *t*_(131)_ = - 0.920, *p* = 0.057, *M*_Exp_ = 22.61, *SD*_Exp_ = 3.89, and *M*_Con_ = 23.89, *SD*_Con_ = 3.78). Participants gave written informed consent according to the guidelines of the ethical standards of the Declaration of Helsinki and were told that they could end the experiment at any time. All procedures were approved by the ethical review board of the Faculty of Human Sciences at the University of Bern.

### Attention Training Tasks

**Figure 1:**
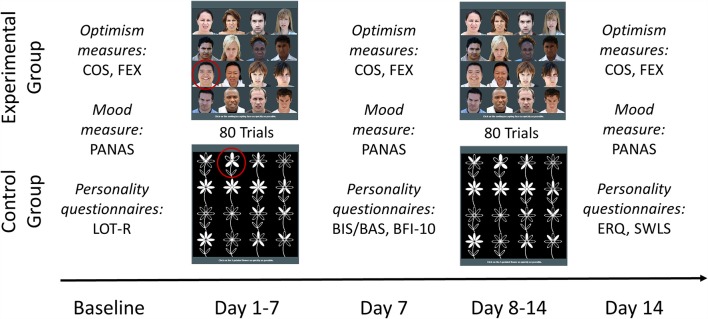
Schematic sequence of the experimental procedure. At baseline, all participants completed the Comparative Optimism Scale (COS; Weinstein, 1980) and Future Expectancy Scale (FEX; Peters et al., 2015) as optimism bias measures (primary outcome), the Positive and Negative Affect Schedule (PANAS; Watson et al., 1988) as a mood measure (secondary outcome), and the Life Orientation Test-Revised (LOT-R; Scheier et al., 1994). For the following 14 days, participants completed daily 80-trial attention bias modification training (ABMT) or control attention training. During ABMT, participants were instructed to click as quickly as possible on the smiling face (here circled in red) among 15 frowning faces in a 4-by-4 matrix; during control training, participants were instructed to click as quickly as possible on the 5-petaled flower (here circled in red) among 15 7-petaled flowers (based on Dandeneau and Baldwin, 2004). On day 7, all participants completed the optimism bias/mood measures, the Behavioral Inhibition System/Behavioral Activation System Scales (BIS/BAS; Carver and White, 1994) and the 10-Item Big Five Inventory (BFI-10; Rammstedt, 2007). On day 14, all participants completed the optimism bias/mood measures, the Emotion Regulation Questionnaire (ERQ; Gross and John, 2003), and the Satisfaction with Life Scale (SWLS; Diener et al., 1985). BIS/BAS, BFI-10, ERQ, and SWLS have been conducted for a larger project on individual differences associated with optimism bias.

Stimuli in the experimental training task (ABMT) comprised colored photographs of a smiling/accepting and a frowning face of 16 different people (half female) that were taken from a larger stimulus set collected at Mark Baldwin’s “Social Cognition and Social Intelligence Lab” at McGill University. Stimuli were presented on a 4-by-4 matrix that appeared in the top middle of the participants’ computer screen. Each matrix displayed one accepting face (target stimulus) and 15 frowning faces (distractor stimuli; see Figure 1)^1^. Participants were instructed to click as quickly as possible with their computer mouse on the accepting face. Stimuli appeared at a random location within the matrix in each of the 80 training trials. Every trial was presented until the participant had clicked on the target and the next trial followed (no inter-trial interval). Difficulty of the training task did not adapt to participants’ performance and participants did not receive feedback on their performance during the task.

Stimuli in the control task comprised black and white drawings of five- and seven-petaled flowers. The procedure in the control task was identical to that of the experimental task, except that each matrix displayed one five-petaled and 15 seven-petaled flowers and participants were instructed to click on the five-petaled flower as quickly as possible. Thus, the task controlled for activity of engaging in a visual search (while not directing attention toward smiling or away from frowning faces).

### Procedure

Participants were told that the study’s purpose was to investigate training to improve responsiveness. For 2 weeks, they performed daily 5-min online training on their computer and indicated whether they had performed the training completely, partly, or not at all on an online questionnaire. Moreover, participants completed personality questionnaires (see Supplementary Appendix A) before, 1 week after, and 2 weeks after training began (to prevent suggestibility effects, participants were not informed about different training versions or that effects on optimism were being investigated). Participants received a daily e-mail message containing links to their version of the training and questionnaires. If participants had not answered the questionnaires by that evening, they were reminded. After the last training, participants were debriefed.

### Dependent Variables

#### Primary Outcome (Optimism Regarding Future Positive Events)

On the COS (Weinstein, 1980), participants indicated the likelihood of themselves, compared to another person of the same age and gender, to experience 18 positive (e.g., “Marrying someone wealthy”) and 23 negative future life events (e.g., “Having a heart attack”) on a scale ranging from -3 (much less likely) to 3 (much more likely)^2^. On the FEX (Peters et al., 2015), measuring state optimism, participants indicated the likelihood of experiencing 10 positive (e.g., “You will get a lot of satisfaction out of life”) and 10 negative future events (e.g., “You will have health problems”) on a 7-point Likert scale ranging from 1 (“not at all likely to occur”) to 7 (“very likely to occur”). Sub-scores representing comparative optimism bias and state optimism about future positive events were computed by using mean scores of participants’ answers to positive items of the COS and FEX. Reliability was acceptable for the positive subscale of the COS (Cronbach’s *α* = 0.71) and good for the positive subscale of the FEX (Cronbach’s *α* = 0.85) in the current sample.

#### Secondary Outcome (Mood)

On the PANAS (Watson et al., 1988), participants indicated how strongly they experienced 10 positive (e.g., “excited”) and 10 negative feelings (e.g., “distressed”) at the moment on a 5-point scale ranging from 1 (“not at all”) to 5 (“very much”). Sub-scores representing positive and negative mood were computed by using sum scores of participants’ answers to positive and negative items of the PANAS. In the current sample, reliability was good for both the positive (Cronbach’s *α* = 0.85) and the negative subscale of the PANAS (Cronbach’s *α* = 0.81).

#### Exploratory Outcomes (Optimism Regarding Future Negative Events)

Additionally, sub-scores representing comparative optimism bias and state optimism about future negative events were computed by using mean scores of participants’ answers to negative items of the COS and FEX for an exploratory analysis. Reliability was good for the negative subscale of the COS (Cronbach’s *α* = 0.88) and acceptable for the negative subscale of the FEX (Cronbach’s *α* = 0.78) in the current sample.

### Data Analysis

#### Primary Outcome (Optimism Regarding Future Positive Events)

We hypothesized that performing positive-search ABMT increases comparative optimism bias and state optimism for future positive events, whereas performing neutral control training does not. We performed two 3 × 2 analyses of variance (ANOVAs) with the within-subject factor time (baseline, one training week, two training weeks) and the between-subject factor group (experimental, control) on positive sub-scores of COS (Weinstein, 1980) and FEX (Peters et al., 2015). Support for our hypothesis should be reflected in significant time × group interactions. To ensure that potential effects on comparative optimism bias and state optimism cannot be explained by group differences in trait optimism, participants’ trait optimism scores were included as a covariate in the analyses.

#### Secondary Outcome (Mood)

We also performed two 3 × 2 ANOVAs with the within-subject factor time (baseline, one training week, two training weeks) and the between-subject factor group (experimental, control) on positive and negative sub-scores of the PANAS (Watson et al., 1988). However, we did not hypothesize, in consistency with earlier findings, an effect of either the positive-search ABMT or the neutral control training on positive or negative mood.

#### Exploratory Outcomes (Optimism Regarding Future Negative Events)

We further explored whether performing the positive-search ABMT or the neutral control task influenced comparative optimism bias and state optimism regarding future negative events. In this context, we also wanted to test the degree to which effects observed for positive events are comparable to those observed for negative events (for both optimism bias and state optimism). Therefore, we performed two 3 × 2 × 2 ANOVAs with the within-subject factors time (baseline, one training week, two training weeks) and valence (future positive events, future negative events) and the between-subject factor group (experimental, control). Participants’ trait optimism scores were included as a covariate in the analyses. We additionally performed two 3 × 2 ANOVAs with the within-subject factor time (baseline, one training week, two training weeks) and the between-subject factor group (experimental, control) on negative sub-scores of COS (Weinstein, 1980) and FEX (Peters et al., 2015). Again, participants’ trait optimism scores were included as a covariate in the analyses.

Significant interactions were further investigated by *post hoc* (Sidak corrected) pairwise comparisons. An α-level of 0.05 (two-tailed) was applied to all analyses. Reported effect sizes are partial eta-squared and noted as $\eta_{\text{p}}^{2}$. If the sphericity assumption was violated, Greenhouse-Geisser corrected values are reported.

## Results

### Training Adherence

On average^3^, participants completed 13 of 14 training sessions and training adherence did not differ between the experimental and control groups (*t*_(145)_ = 0.770, *p* = 0.442, *M*_Exp_ = 13.05, *SD*_Exp_ = 2.41, and *M*_Con_ = 12.76, *SD*_Con_ = 2.22). Of the 147 participants who initially enrolled in the study and had no technical errors during data collection, 81 (55.1%) completed all 14 training sessions, 38 (25.9%) completed 13 of 14 training sessions, 13 (8.8%) completed 12 of 14 training sessions, and one completed 11 of 14 training sessions and started the other three training sessions without finishing (totaling the 133 participants included in the analysis). The remaining 14 participants (9.5%) completed 1 (*N* = 2), 2 (*N* = 2), 4 (*N* = 1), 8 (*N* = 1), 9 (*N* = 1), or 11 (*N* = 8) of the 14 training sessions.

### Primary Outcome (Optimism Regarding Future Positive Events)

**Figure 2:**
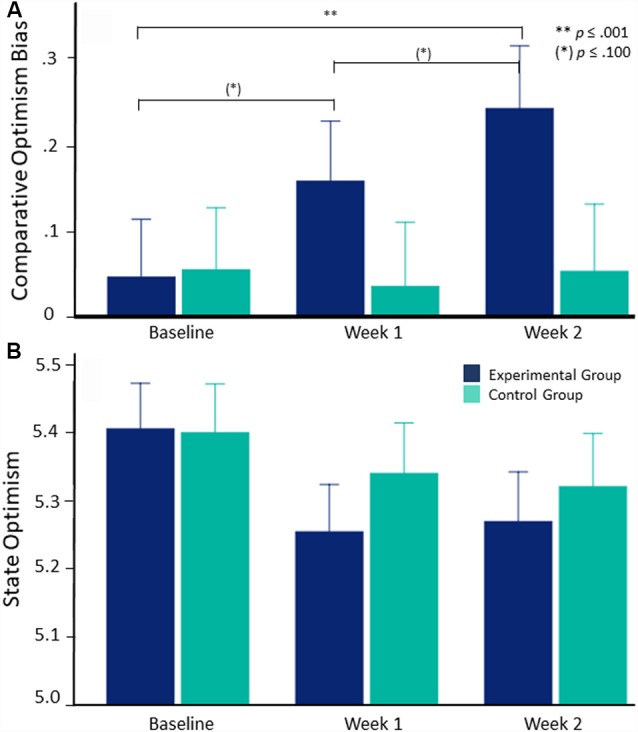
Change in comparative optimism bias and state optimism from baseline to after two training weeks in the experimental/control group. Error bars depict standard errors. **(A)** Comparative optimism bias significantly increases over the 2-week training period in the experimental group but does not change in the control group when we control for trait optimism. **(B)** State optimism does not differ between groups or change over time when we control for trait optimism.

Comparative optimism bias regarding future positive events did not generally differ between groups, *F*_(1, 130)_ = 1.119, *p* = 0.292, $\eta_{\text{p}}^{2}$ = 0.009, or change over time, when we controlled for variations in trait optimism, *F*_(2, 227)_ = 0.295, *p* = 0.714, $\eta_{\text{p}}^{2}$ = 0.002. Notably, the predicted time × group interaction was significant when we controlled for variations in trait optimism, *F*_(2, 227)_ = 4.339, *p* = 0.018, $\eta_{\text{p}}^{2}$ = 0.032. In line with our hypothesis, comparative optimism bias regarding future positive events increased from before to after two training weeks and showed a trend to increase from before to after one training week and from after one to after two training weeks when people performed daily ABMT (baseline vs. two training weeks: *p* = 0.001, baseline vs. one training week: *p* = 0.070, one training week vs. two training weeks: *p* = 0.066, as revealed by *post hoc* pairwise comparisons). Comparative optimism bias regarding future positive events did not change when people performed neutral control training (baseline vs. two training weeks: *p* = 1.000, baseline vs. one training week: *p* = 0.976, one training week vs. two training weeks: *p* = 0.958; see Figure 2A).

By contrast, state optimism regarding future positive events did not differ between groups, main effect of group, *F*_(1, 130)_ = 0.218, *p* = 0.641, $\eta_{\text{p}}^{2}$ = 0.002; time × group interaction, *F*_(2, 226)_ = 0.758, *p* = 0.453, $\eta_{\text{p}}^{2}$ = 0.006, or change over time when we controlled for variations in trait optimism, *F*_(2, 226)_ = 0.482, *p* = 0.591, $\eta_{\text{p}}^{2}$ = 0.004 (Figure 2B).

### Secondary Outcome (Mood)

**Figure 3:**
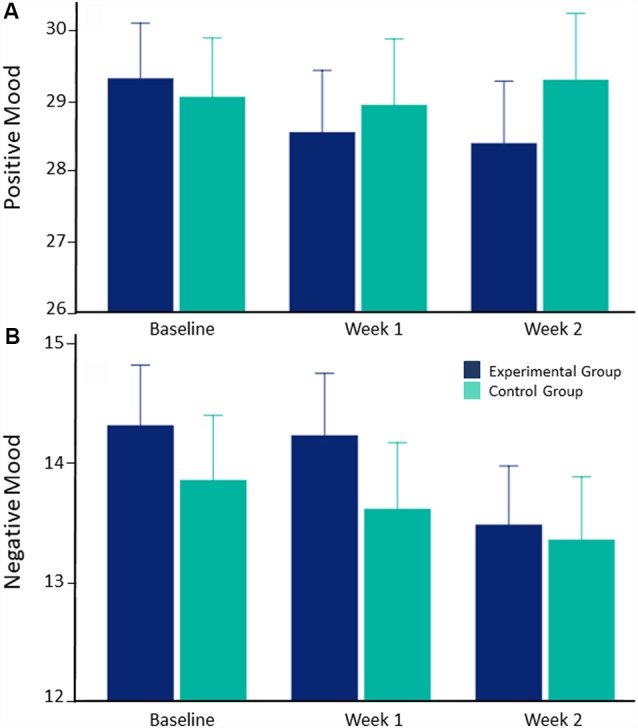
Change in positive and negative mood from baseline to after two training weeks in the experimental/control group. Error bars depict standard errors. **(A)** Positive mood does not change over the 2-week training period in the experimental or control group. **(B)** Negative mood does not change over the 2-week training period in the experimental or control group.

Positive mood did not differ between groups, main effect of group, *F*_(1, 131)_ = 0.095, *p* = 0.759, $\eta_{\text{p}}^{2}$ = 0.001; time × group interaction, *F*_(2, 262)_ = 0.671, *p* = 0.512, $\eta_{\text{p}}^{2}$ = 0.005, or change over time, *F*_(2, 262)_ = 0.418, *p* = 0.659, $\eta_{\text{p}}^{2}$ = 0.003 (Figure 3A). Similarly, negative mood did not differ between groups, main effect of group, *F*_(1, 131)_ = 0.377, *p* = 0.540, $\eta_{\text{p}}^{2}$ = 0.003; time × group interaction, *F*_(2, 232)_ = 0.313, *p* = 0.705, $\eta_{\text{p}}^{2}$ = 0.002, or change over time, *F*_(2, 232)_ = 2.423, *p* = 0.091, $\eta_{\text{p}}^{2}$ = 0.018 (Figure 3B).

### Exploratory Outcomes (Optimism Regarding Future Negative Events)

An exploratory analysis revealed that when valence was added as an additional factor in the analysis, there was only a marginally significant time × valence × group interaction, *F*_(2, 220)_ = 2.903, *p* = 0.066, $\eta_{\text{p}}^{2}$ = 0.022, regarding comparative optimism bias for future events when we controlled for variations in trait optimism. Furthermore, comparative optimism bias regarding future negative events did not differ between groups, main effect of group, *F*_(1, 130)_ = 0.2.326, *p* = 0.130, $\eta_{\text{p}}^{2}$ = 0.018; time × group interaction, *F*_(2, 222)_ = 0.023, *p* = 0.964, $\eta_{\text{p}}^{2}$ = 0.000, or change over time, *F*_(2, 222)_ = 0.040, *p* = 0.961, $\eta_{\text{p}}^{2}$ = 0.000, when we controlled for variations in trait optimism.

There was no significant time × valence × group interaction, *F*_(2, 208)_ = 1.290, *p* = 0.277, $\eta_{\text{p}}^{2}$ = 0.010, regarding state optimism regarding future events when we controlled for variations in trait optimism. State optimism regarding future negative events was significantly lower in the experimental group than in the control group when we controlled for trait optimism, main effect of group *F*_(1, 130)_ = 4.997, *p* = 0.027, $\eta_{\text{p}}^{2}$ = 0.037. However, there was no time × group interaction, *F*_(2, 225)_ = 0.674, *p* = 0.265, $\eta_{\text{p}}^{2}$ = 0.010, and state optimism regarding future negative events did not change over time, *F*_(2, 225)_ = 1.062, *p* = 0.340, $\eta_{\text{p}}^{2}$ = 0.008, when we controlled for variations in trait optimism.

## Discussion

The present experiment demonstrates that repeatedly directing attention toward smiling faces and away from frowning faces over 2 weeks enhances comparative optimism bias for future positive events, whereas performing neutral control attention training does not (Weinstein, 1980). Adherence to the online attention training used in the present study was generally high (about 90% of participants completed all or the great majority of training sessions). Furthermore, enhanced optimism bias was specific to future positive events and could not be attributed to peoples’ mood (i.e., positive and negative feelings did not change over the training period) or be explained by individual differences in trait optimism (which was controlled for in the analyses).

Thus, training a cognitive habit to pay attention to positive social information not only increases self-esteem and reduces stress, but also enhances optimism bias, an important protective factor for mental health (Dandeneau et al., 2007; Sharot, 2011). What is more, this finding supports the combined cognitive biases hypothesis and implies that (a) expectancy biases are an essential part of the hypothesis (despite being rarely considered in past research; Aue and Okon-Singer, 2015); and (b) cognitive bias interactions are not only present in psychological disorders, but also extend to positivity biases in healthy individuals (Kress and Aue, 2017).

Notably, performing the ABMT does not increase state optimism, but has specific effects on comparative optimism bias. There are two possible explanations for this distinction. First, items of the FEX (Peters et al., 2015) used to measure state optimism are more general than items of the COS (Weinstein, 1980) and might therefore uncover temporary variations in dispositional optimism (i.e., a general positive life orientation that is not necessarily biased, such as the belief that good things will happen) rather than in optimism bias (i.e., biased expectancies about the likelihood of specific future life events, such as being more likely than other people to live past 85 years). Even though dispositional optimism might increase one’s readiness to display optimism bias, the two phenomena represent separate concepts (Shepperd et al., 2015).

Second, it is possible that the ABMT used in the current study specifically influenced self-enhancing aspects of comparative optimism bias related to social comparison (Hoorens, 1996). The ABMT has been shown to increase self-esteem (Dandeneau and Baldwin, 2004), which plays an important role in social comparison (Jones and Buckingham, 2005) and may, therefore, mediate the relation between positive attention processes and comparative optimism bias. Furthermore, the ABMT’s social stimuli may have had specific effects on the strong social component of comparative optimism bias (i.e., social comparison). A more general ABMT (e.g., using specific words that do not convey a strong social component) might also influence state optimism. To draw final conclusions on such mediating factors, future research should directly examine the relationship between social and non-social ABMT, different measures of optimism bias, and self-esteem.

Notably, we found that, in accordance with the postulate that optimism biases for positive and negative future events represent different aspects with independent motivating factors (Weinstein, 1980; Hoorens, 1996), the influence of the ABMT on comparative optimism was limited to the positive events^4^. Our exploratory analyses for the negative events revealed solely a main effect of group regarding state optimism. However, because this effect remained stable across the three time points considered (i.e., existed already before the experiment), the effect cannot be attributed to the training. It remains to be determined whether other attention modification procedures (e.g., those that train the individual to shift attention away from negative stimuli) are more effective in modifying optimism bias for negative events.

Furthermore, specific mechanisms driving the behavioral effect reported in the current study could be revealed by investigating its underlying neural correlates. Prior investigations of the neural correlates underlying threat-avoidance ABMT, in which people train to direct their attention *away* from negative, maladaptive information, have shown that ABMT may *reduce* activity in limbic brain areas such as the amygdala and insula (Månsson et al., 2013; Taylor et al., 2014) and enhance frontal control (Browning et al., 2010; Taylor et al., 2014). In contrast, positive-search ABMT, in which people train to direct their attention *toward* positive, adaptive information, could make positive social information (i.e., happy faces) more salient and therefore *increase* amygdala and insula activity. Because people usually base their expectancies about the future on information they currently have at hand, we have previously suggested that biased attention toward positive environmental information could strengthen optimism bias and that this process is supported by specific activations in parietal and cingulate cortices (Kress and Aue, 2017). By increasing the saliency of positive, adaptive information, positive-search ABMT could facilitate bottom-up attentional shifts to similar adaptive information in people’s environment, and—over time—strengthen optimism bias regarding the future. Of note, the brain’s saliency network (comprising the insula and the dorsal anterior cingulate cortex) and the executive control network (especially its more parietal brain areas) have already been shown to play a crucial role in the reverse causal effect, namely, when optimistic expectancies guide attention to positive information (Kress et al., under revision).

Three methodological features of this work might limit the conclusions to be drawn. First, we chose online training in the current study to make sure the training could be easily administered on a large scale (which is the eventual purpose of such cognitive training) and would, therefore, be more useful in both a clinical and non-clinical setting (Holmes et al., 2018). Unfortunately, we were not able to monitor how often participants performed the online training; thus, information on training adherence was based on participants’ self-report. However, social desirability bias is usually reduced when questionnaires are self-administered online (Nederhof, 1985) and there is no reason to suspect that social desirability bias would differ between participants in the experimental and control conditions. What is more, if study adherence had been lower than proclaimed by the participants, the observed differences between the experimental conditions should have been even larger. Thus, the main finding observed in the current study (i.e., the influence of the positive ABMT training on comparative optimism bias) should not be limited by self-reported adherence data.

Second, the control training used in the current study did not contain face stimuli (as did the ABMT) and therefore does not control for exposure to faces (and potential associated social effects). For better comparison with earlier findings, we decided to use the same conditions as in prior research on positive-search ABMT (Dandeneau and Baldwin, 2004, 2009; Dandeneau et al., 2007; De Voogd et al., 2014, 2016). Of note, Dandeneau et al. (2007) did include an additional control condition in which participants were asked to look at a matrix of frowning faces similar to the one used in the ABMT. Whereas the ABMT did modulate attention to acceptance/rejection information, pure stimulus exposure did not, making it unlikely that exposure to face stimuli drove beneficial effects on optimism bias in the current study. Yet, to securely rule out this alternative interpretation, future research could include an additional “social” control condition, in which participants are exposed to face stimuli but have to search for a different feature (e.g., the face with brown hair/eyes rather than the smiling face).

The third potential shortcoming relates to the fact that we did not assess whether and how ABMT changed attention processes in the current study (e.g., whether attention bias or attentional control changed throughout the training). Because previous research has already shown that the specific positive-search ABMT used in the current study changes attention bias (Dandeneau et al., 2007), we focused on the training’s outcome (i.e., whether extensively training a cognitive habit to direct attention to positive information enhances self-reported optimism bias) instead of the exact attentional mechanisms causing this outcome. Thus, even though it is most likely that the attentional processes targeted by the training instigated changes in optimism bias, we cannot exclude the possibility that other mechanisms contributed to the reported training effects.

One such mechanism could be stimulus exposure. As mentioned above, because the control training used in the current study did not contain face stimuli, it is possible that changes in optimism bias caused by the ABMT were partly due to stimulus exposure to frowning and smiling faces. However, the ABMT contained an overwhelming majority of frowning faces (each array consisted of 15 frowning faces and only one smiling face), which should from a theoretical point of view reduce rather than increase optimism (Kress and Aue, 2017). Moreover, prior research that used the exact same training protocol as the current study revealed that effects on self-reported outcome measures (e.g., self-esteem) are not merely due to stimulus exposure but instead rely on active attentional mechanisms (Dandeneau et al., 2007). Yet, it is crucial that future research replicates the current findings and additionally includes attention measures to shed light on the exact mechanisms leading to the training’s effects on optimism bias.

The specificity of traditional threat-avoidance ABMT in modifying attention bias and emotional outcomes has been discussed controversially because (1) control trainings have often elicited similar changes, and (2) threat-avoidance ABMT may also affect other aspects of attention such as attentional control (Heeren et al., 2015a). Following these controversies, it has been suggested to instead adapt ABMT on the basis of theoretical considerations and investigate its benefits for emotional outcomes (Mogg and Bradley, 2018). When such novel ABMT approaches reliably elicit emotional benefits, attentional mechanisms potentially underlying these benefits should be investigated with multiple measures (e.g., for initial attention orienting, attention maintenance, attention bias variability, attentional control; see Mogg and Bradley, 2016; Mogg et al., 2017; Waters et al., 2016). Notably, from a theoretical perspective, both controlled top-down and automatic bottom-up attention processes potentially targeted by the ABMT are relevant for the mutually enforcing optimism-attention interactions that we aimed to investigate (Kress and Aue, 2017). To draw final conclusions about exactly which attentional mechanisms cause benefits of positive-search ABMT on optimism bias, future research needs to investigate (a) how training affects multiple attentional processes and (b) how this relates to changes in optimism bias. Such investigation can then further refine positive-search ABMT to make it more effective.

Subsequent studies should further include the collection of performance data to (i) control for potential differences in training difficulty/performance between the ABMT and control training and (ii) investigate the influence of different combinations of individual performance and training types on optimism in a dose-response manner. Furthermore, comparative optimism bias could be measured indirectly (i.e., by asking participants to rate the probability of a positive future event happening to them and happening to another person separately) to reveal additional information on whether the ABMT training increased ratings for themselves and/or decreased ratings for the average other. The specific assessment of comparative optimism in the current study did not permit such conclusions because participants rated their personal likelihood of encountering positive future events related to the likelihood of the average person of the same gender and age.

In general, the present findings contribute to a more nuanced view on the cognitive processes underlying optimism bias. A cognitive habit to pay attention to positive information is likely involved in the development and maintenance of optimism bias and, therefore, reveals how it can be triggered and maintained (Kress and Aue, 2017).

We have previously shown that optimistic expectancies strongly guide attention toward reward (Kress et al., 2018) and hypothesized that subsequent attention to positive information stabilizes optimism bias. Such supportive attention processes could explain why future expectancies are selectively updated into an optimistic (not a pessimistic) direction following feedback (Sharot, 2011). The current results independently reveal the crucial missing piece of information corroborating our idea that attention processes maintain optimism bias over time: Directing attention to positive information does indeed enhance optimism bias and can thereby provoke positive feedback effects on initial optimistic expectancies. Together, these findings argue for dynamic bidirectional optimism-attention interactions that maintain positivity and contribute to well-being and mental health.

Identifying the concrete attentional mechanisms underlying optimism bias will contribute in an important way to our understanding of its maintenance over time. Along these lines, future research should uncover the neural basis underlying this optimism-attention interplay, thereby supplementing and informing behavioral investigations. Specifically, the neurocognitive model proposed to underlie the dynamic optimism-attention interplay (Kress and Aue, 2017) needs to be backed up with further empirical neural data. For instance, it is possible that optimistic expectancies drive ongoing visual attention toward supporting positive information *via* top-down mechanisms initiated in frontal and prefrontal brain regions (e.g., anterior cingulate cortex, ventromedial prefrontal cortex/orbitofrontal cortex). At the same time, it is possible that bottom-up attentional shifts toward positive environmental information represented in more posterior and parietal brain regions (e.g., posterior parietal cortex, posterior cingulate cortex) strengthen optimism about the future (as future expectations are usually based on information currently at hand).

Examining the neural correlates of positive-search ABMT influencing optimism bias can thus provide essential information on which brain regions are activated when shifts in attention influence optimism and additionally extend the existing literature on neural mechanisms of ABMT which has, so far, focused on threat avoidance (Browning et al., 2010; Månsson et al., 2013; Taylor et al., 2014). Moreover, neuroimaging studies can identify brain areas involved in dynamic cognitive-bias interactions and point to the neurotransmitter systems that are involved. In case an individual manifests malfunctioning or maladaptive interactions (i.e., in psychological disorders), these might then be targeted by specific cognitive and pharmacological interventions. Studies examining neural correlates of cognitive-bias interactions may, therefore, reveal valuable insights for applications in the clinical domain and in everyday life.

In fact, a central finding in the current study is that (comparative) optimism bias is malleable and can be easily modified by adequate attention modification procedures. Thus, being optimistic can be learned or trained and one is not deemed to be either high or low on optimism bias. Moreover, the present findings imply that, in everyday life, focusing on positive aspects of the environment can boost optimism, and thereby most certainly motivation, concerning a difficult task. In the clinical domain, the findings imply that changing one aspect of biased cognition can alter other aspects, thereby revealing multiple starting points for possible modification. The current evidence is hence suggestive and might improve overall conditions for the prevention and treatment of psychological disorders. Notably, ABMT has especially great potential because it can be a low-cost, standardized, and easy-to-access support for psychotherapy. Online training that does not require therapist contact constitutes a first intervention for people with contact anxiety (e.g., social phobia) and for patients who have to wait months before seeing a psychotherapist because of an overstrained health system.

In conclusion, our data show that directing attention toward positive and away from negative social information enhances comparative optimism bias. Uncovering such cognitive processes underlying optimism bias is essential for employing its benefits for mental health. Positive-search ABMT could trigger a self-sustaining upward spiral of positivity (through dynamic optimism-attention interactions), making our findings central for individual well-being as well as for the prevention and treatment of psychological disorders (Garland et al., 2010; Kress and Aue, 2017). In particular, the present findings reveal that paying attention to positive information around us makes us more optimistic about our future and they lead to some practical advice: If we want to look toward a great future, we should start looking at the good things around us right now.

## Ethics Statement

Participants gave written informed consent according to the guidelines of the ethical standards of the Declaration of Helsinki and were told that they could end the experiment at any time. All procedures were approved by the local ethical review board.

## Author Contributions

LK and TA designed and planned the experiment and reviewed and edited the manuscript. LK performed the experiment, and analyzed the data, and wrote the initial draft of the manuscript. TA provided funding and supervised the project.

## Conflict of Interest Statement

The authors declare that the research was conducted in the absence of any commercial or financial relationships that could be construed as a potential conflict of interest.

## Footnotes

^1^The attention bias modification training and control training used in the current study can be accessed *via* the following links: ABMT-http://baldwinlab.mcgill.ca/labmaterials/materials_16fa_c_80.html; Control training- http://baldwinlab.mcgill.ca/labmaterials/materials_16fl_80.html

^2^The German in-house translation of the COS (Weinstein, 1980) used in the current study can be found in Supplementary Appendix B.

^3^The raw data of this study can be found in Supplementary Appendix C.

^4^Note, however, that there was only a marginally significant three-way interaction between the factors time, valence of the event, and group in the three-factorial ANOVA comparing effects for positive vs. negative events; thus the specificity of the ABMT’s effects on comparative optimism bias for positive future events needs to be further investigated.
