# Supplementary material for: Learning to Look at the Bright Side of Life: Attention Bias Modification Training Enhances Optimism Bias
Source: Front Hum Neurosci. 2019 Jul 9;13:222. doi: 10.3389/fnhum.2019.00222 (PMC6629951; doi:10.3389/fnhum.2019.00222)
Supplement: Supplementary file 1 [file Appendix_A.docx]

**Appendix A**

Learning to look at the bright side of life: Attention bias modification training enhances optimism bias

Laura Kress and Tatjana Aue

# Appendix A. List of personality questionnaires participants completed during the study.

Life Orientation Test – Revised (LOT-R; Scheier et al., 1994)

Behavioral Inhibition System/Behavioral Activation System Scales (BIS/BAS; Carver & White, 1994)

10-Item Big Five Inventory (BFI-10; Rammstedt, 2007)

Emotion Regulation Questionnaire (ERQ; Gross & John, 2003)

Satisfaction with Life Scale (SWLS; Diener, Emmons, Larsen, & Griffin, 1985)

# References

Carver, C. S., & White, T. L. (1994). Behavioral inhibition, behavioral activation, and affective responses to impeding reward and punishment: The BIS/BAS Scales. *Journal of Personality and Social Psychology*, *67*(2), 319–333.

Diener, E., Emmons, R. A., Larsen, R. J., & Griffin, S. (1985). The Satisfaction with Life Scale. *Journal of Personality Assessment*, *49*(1), 71–75. https://doi.org/10.1207/s15327752jpa4901_13

Gross, J. J., & John, O. P. (2003). Individual differences in two emotion regulation processes: implications for affect, relationships, and well-being. *Journal of Personality and Social Psychology*, *85*(2), 348–362.

Rammstedt, B. (2007). The 10-Item Big Five Inventory - Norm values and investigation of sociodemographic effects based on a German population representative sample. *European Journal of Psychological Assessment*, *23*(3), 193–201. https://doi.org/10.1027/1015-5759.23.3.193

Scheier, M. F., Carver, C. S., & Bridges, M. W. (1994). Distinguishing optimism from neuroticism (and train anxiety, self-mastery, and self-esteem): A reevaluation of the life orientation test. *Journal of Personality and Social Psychology*, *67*(6), 2063-1078. http://dx.doi.org/10.1037/0022-3514.67.6.1063
