# Supplementary material for: Learning to Look at the Bright Side of Life: Attention Bias Modification Training Enhances Optimism Bias
Source: Front Hum Neurosci. 2019 Jul 9;13:222. doi: 10.3389/fnhum.2019.00222 (PMC6629951; doi:10.3389/fnhum.2019.00222)
Supplement: Supplementary file 2 [file Appendix_B.docx]

**Appendix B**

Learning to look at the bright side of life: Attention bias modification training enhances optimism bias

Laura Kress and Tatjana Aue

**Appendix B.** German translation of the Comparative Optimism Scale (COS; Weinstein, 1980) used in the current study. The 42 items were presented in random order and rated on a 7-point scale ranging from -3 (“much less likely”/German version: “viel unwahrscheinlicher”) to 3 (“much more likely”/German version: “viel wahrscheinlicher”). Items of the COS were translated into German using a standard forward-backward translation procedure.

Verglichen mit einer Person Ihres Alters und Ihres Geschlechts, was denken Sie, wie hoch ist Ihre Wahrscheinlichkeit...

... einen Job zu bekommen?

... ein eigenes zu Haus besitzen?

... einen Einstiegslohn über 7'000 CHF pro Monat zu bekommen?

... auf einen anderen Kontinent zu reisen?

... einen Einstiegslohn über 9'000 CHF pro Monat zu bekommen?

... ein gutes Jobangebot vor Ausbildungsabschluss zu bekommen?

... zum besten Drittel der Absolventen Ihres Studiengangs zu gehören?

... ein Eigenheim zu besitzen, das seinen Wert innerhalb der nächsten 5 Jahre verdoppelt?

... eine Auszeichnung für Ihre Arbeit zu erhalten?

... über 85 Jahre alt zu werden?

... wegen Ihrer Leistungen in eine Zeitung zu kommen?

... in den nächsten 5 Jahren keine Nacht im Krankenhaus zu verbringen?

... einmal ein hochbegabtes Kind zu haben?

... landesweite Anerkennung für Ihre Arbeit zu bekommen?

... Ihr Gewicht in den nächsten 10 Jahren konstant zu halten?

... in den nächsten 10 Jahren mehr als 25'000 CHF pro Monat zu verdienen?

... im Winter nie krank zu werden?

... jemanden mit grossem Vermögen zu heiraten?

... ein Alkoholproblem zu haben?

... Suizid zu versuchen?

... sich wenige Jahre nach einer Heirat scheiden zu lassen?

... einen Herzinfarkt vor dem 40. Lebensjahr zu haben?

... eine Geschlechtskrankheit zu bekommen?

... von einem Arbeitgeber gefeuert zu werden?

... Lungenkrebs zu bekommen?

... unfruchtbar zu werden?

... Ihre Ausbildung abbrechen zu müssen?

... einen Herzinfarkt zu bekommen?

... 6 Monate lang keinen Job zu finden?

... einen Zahn aufgrund von Kariesbefall ziehen lassen zu müssen?

... Zahnfleischprobleme zu haben?

... einen unattraktiven Job annehmen zu müssen?

... eine Fehlentscheidung beim Autokauf zu treffen?

... festzustellen, den falschen Karriereweg gewählt zu haben?

... zu stolpern und sich einen Knochen zu brechen?

... von jemandem verklagt zu werden?

... Opfer eines Autodiebstahls zu werden?

... Opfer eines Überfalls zu werden?

... an Krebs zu erkranken?

... für 2 oder mehr Tage krank im Bett zu liegen?

... Opfer eines Einbruchs zu werden?

... bei einem Autounfall verletzt zu werden?
